# Some aspects of purinergic signaling in the ventricular system of porcine brain

**DOI:** 10.1186/1751-0147-53-54

**Published:** 2011-10-13

**Authors:** Joanna Czarnecka, Katarzyna Roszek, Artur Jabłoński, Dariusz Jan Smoliński, Michał Komoszyński

**Affiliations:** 1 Biochemistry Department, Institute of General and Molecular Biology, Nicolaus Copernicus University, 7 Gagarina St, 87-100 Torun, Poland; 2 Department of Swine Diseases, National Veterinary Research Institute, 57 Partyzantow Avenue, 24-100 Pulawy, Poland; 3 Cell Biology Department, Institute of General and Molecular Biology, Nicolaus Copernicus University, 7 Gagarina St, 87-100 Torun, Poland

**Keywords:** extracellular nucleotides, ecto-nucleotidases, exo-nucleotidases, nucleotide receptor, brain ventricular system

## Abstract

**Background:**

Numerous signaling pathways function in the brain ventricular system, including the most important - GABAergic, glutaminergic and dopaminergic signaling. Purinergic signalization system - comprising nucleotide receptors, nucleotidases, ATP and adenosine and their degradation products - are also present in the brain. However, the precise role of nucleotide signalling pathway in the ventricular system has been not elucidated so far. The aim of our research was the identification of all three elements of purinergic signaling pathway in the porcine brain ventricular system.

**Results:**

Besides nucleotide receptors on the ependymocytes surface, we studied purines and pyrimidines in the CSF, including mechanisms of nucleotide signaling in the swine model (*Sus scrofa domestica*). The results indicate presence of G proteins coupled P2Y receptors on ependymocytes and also P2X receptors engaged in fast signal transmission. Additionally we found in CSF nucleotides and adenosine in the concentration sufficient to P receptors activation. These extracellular nucleotides are metabolised by adenylate kinase and nucleotidases from at least two families: NTPDases and NPPases. A low activity of these nucleotide metabolising enzymes maintains nucleotides concentration in ventricular system in micromolar range. ATP is degraded into adenosine and inosine.

**Conclusions:**

Our results confirm the thesis about cross-talking between brain and ventricular system functioning in physiological as well as pathological conditions. The close interaction of brain and ventricular system may elicit changes in qualitative and quantitative composition of purines and pyrimidines in CSF. These changes can be dependent on the physiological state of brain, including pathological processes in CNS.

## Background

Ventricular system is composed of brain structures lined with ependyma and is filled with cerebrospinal fluid (CSF). The composition and physico-chemical properties of CSF depend on the physiological condition of brain. In turn, the composition of CSF influences the function of brain cells. It is well known that cerebrospinal fluid transports signaling molecules and trophic factors generating complex physiological responses due to the activation of their appropriate receptors present on the cells contacting the CSF [1-4]

There are numerous signalization pathways functioning in the brain ventricular system. The most important are GABAergic, glutaminergic and dopaminergic signaling [5-11]. Elements of purinergic signalization system - nucleotide receptors, nucleotidases, ATP and adenosine and their degradation products - are also present in that brain structure [1-4,12-16].

Purines such as adenine and guanine beside committed in neurotransmition and neuromodulation also function as trophic factors [17-19]. Ectopurines are involved in the activation of differentiation and neuritogenesis of precursor cells and neurons. They stimulate synthesis and release of trophic factors in neuronal and glial cells and enhance the effect of growth factors [17,18]. Purines also participate in immunological response due to astrocytes and microglia activation, initiation of inflammatory reactions, apoptosis and necrosis, as well as glial cell proliferation [17,18,20,21]. Extracellular purines bring about signaling or transfer of the information by activating two classes of the receptors: P1-adenosine receptors and P2-nucleotide receptors [19,22]. The activation of P1 and P2 receptors affects metabolic processes, adhesion, motility and proliferation capability of cells [23,24]. The P1 and P2 receptors are colocalized in the most types of cells, where they act antagonistically and regulate the physiological processes [19,22,25-27].

The purines concentration outside the cell depends on the balance between their release from the cells, uptake and extracellular metabolism [19,25]. The purine nucleotides outside the cell are metabolised by ecto-nucleotidases [25,27]. Activity of these enzymes was detected in all examined living organisms: plants, bacteria, animals and human [25].

All known ecto-enzymes controlling the nucleotides concentration belong to several families, differing in origin and mechanism of action. There are four families of nucleotidases: NTPDases (nucleoside triphosphate diphosphohydrolases), NPPases (pyrophosphohydrolases/phosphodiesterases), phosphatases and ecto-5'nucleotidase. The next group of ecto-enzymes involved in regulation of nucleotides concentration outside the cell are nucleotide kinases - the enzymes that transfer the phosphate moiety between nucleotides. Numerous investigations show that ecto-enzymes metabolizing nucleotides outside the cell are involved in termination of nucleotide signaling pathway due to releasing ligands from their receptors [25,28,29]. Additionally, nucleotidases and kinases may produce other secondary messengers like ATP, ADP and adenosine.

According to the previous studies on rodent brain ventricular system, nucleotide receptors P2X_7 _are localized on the surface of cells lining the cerebral ventricles and cells of choroid plexus [16,30]. P2X_2 _mRNA has been found in neurons contacting CSF in rat spinal cord [15]. However, the precise role of nucleotide signaling pathway in the ventricular system has not been elucidated so far. Surprisingly, there are no data demonstrating the extracellular purines metabolism within the ventricular system of mammals other than rodents. There is little information concerning the soluble nucleotidases in CSF [16,31], and virtually nothing is known about ependymal membrane-bound nucleotidases.

The aim of our research was to elucidate if all three elements of purinergic signaling pathway, including nucleotide receptors on the ependymocytes surface, purines and pyrimidines in the CSF and mechanisms of nucleotide signal termination, are present in the porcine brain ventricular system.

## 2. Materials and methods

### 2.1. Reagents

We used the following reagents: ethanolamine, *n*-heptane, KCl, HClO_4 _and EDTA (POCh Gliwice, Poland, p.a. grade), KH_2_PO_4_, K_2_HPO_4_, tetrabutylammonium hydrogen sulphate (TBA) and isocratic methanol (Baker Phillipsburg, USA, HPLC grade). The following nucleotides and nucleosides: Adenosine-5'-triphosphate, adenosine-5'-diphosphate, adenosine-5'-monophosphate, adenosine, guanosine-5'-triphosphate, guanosine-5'-diphosphate, uridine-5'-triphosphate, uridine-5'-diphosphate (98-99% purity, Sigma-Aldrich, Europe) were used as substrates for enzyme activity determination and as HPLC standards.

For molecular biology analyses: First Strand cDNA Synthesis Kit (Fermentas, Lithuania), primary rabbit antibodies - anti-P2X_7_, anti-P2X_2 _and anti-P2Y_2 _(Sigma-Aldrich, Europe), Alexa Fluor 488 secondary anti-rabbit antibody (Molecular Probes, Leiden, Holland) were used.

Primers were synthesized in DNA Sequencing and Oligonucleotide Synthesis Lab (Institute of Biochemistry and Biophysics, Polish Academy of Sciences, Warsaw, Poland).

### 2.2. Materials

Cerebrospinal fluids (CSFs) of healthy swines were collected in Department of Swine Diseases, National Veterinary Research Institute (Puławy, Poland) following ethical procedures laid by ethical committee of the university. CSFs were centrifuged 10 min at 20 000 × *g *to discard the morphotic elements. The supernatant obtained was immediately used to determine enzymatic activity. Portions of the CSFs were cooled to 4°C, frozen and then stored at -80°C. After thawing the CSFs were centrifuged and used to determine nucleotide concentrations.

Porcine brains collected immediately after slaughter, were placed in cold (4°C) isotonic buffer A (35 mM Tris-HCl pH 7.4, 250 mM sucrose, 10 mM glucose) with 4 mM MgCl_2 _and 2 mM CaCl_2_. The tissue from lateral ventricle was cut into slices (1 cm diameter, 0.785 cm^2^) and used to determine membrane bound activity of enzymes from ependymal cells. The fragments of lateral ventricle wall were cut with the use of sharp steel tubing and immobilized in alginate with the ependymal layer directed upside. These fragments of ventrical wall were used to immunohistochemical localisation of nucleotide receptors as well as for expression analysis of nucleotidases.

### 2.3. Qualitative and quantitative analysis of purines and pyrimidines

The already established method of solid phase extraction (SPE) of purines and pyrimidines [32] and high-performance liquid chromatography (HPLC) was and used in the experiments.

### 2.4. Enzymatic activity determination

**Nucleotidases **assay in CSFs was carried out in incubation mixture composed of: isotonic buffer A with 3 mM Mg^2+ ^or 2 mM Ca^2+^, 2.5 mM levamisol (alkaline phosphatase inhibitor) [33], 0.1 mM dipiridamol (adenosine deaminase inhibitor) [34], and 1 or 2 mM nucleotide (ATP, ADP, AMP) as substrate. To distinguish nucleotidases 0.1 mM suramine (NTPDase inhibitor [35]) or 10 μM Ap5A (NPPase and adenylate kinase inhibitor [36,37]) were added to incubation mixture.

**Adenylate kinase **assay in CSFs was carried out in isotonic buffer A containing 3 mM Mg^2+^, 2.5 mM levamisol, 0.1 mM dipiridamol, 0.1 mM suramine (buffer A) and 2 mM ADP as substrate.

Enzymatic reactions were initiated with 20 μl CSF added to 20 μl of pre-warmed incubation mixture. The samples were incubated for 15-120 min at 37°C and reaction was terminated with 20 μl of 1M cold HClO_4_. All tested samples were neutralized with 1M KOH, delipidated by shaking with *n*-heptane (1:5, v/v), centrifuged and analysed for qualitative and quantitative analysis of purines concentration.

Determination of ependymal ecto-enzymes activity was carried out *in situ *on the tissue surface. The tissue slices were incubated for 10-30 min with appropriate incubation mixtures at 37°C. The reaction was terminated by adding 50 μl of incubation mixture to 50 μl of 1M cold HClO_4_. All tested samples were prepared for the analysis of purines concentration as described above.

The effect of divalent ions influence on the activity of porcine brain ventricular system enzymes was determined in the presence of 2 mM Ca^2+ ^and 3 mM Mg^2+^.

The pH optimum of enzymes metabolizing nucleotides in ventricular system was carried out in pH range between 6.0 to 9.0, in isotonic conditions and in the presence of appropriate divalent ions and inhibitors blocking the enzymatic activities of other enzymes.

### 2.5. Protein assay

Protein concentration was determined by the method of Bradford [38] using bovine serum albumin as a standard.

### 2.6. Western blotting

After completing SDS-PAGE and electrophoretic transfer onto the nitrocellulose, the membrane was blocked in 3% BSA in TBS for 1 hour. The membrane was then incubated for 2 hours at room temperature with the rabbit polyclonal primary antibody (anti-NTPDase1 (CD39) antibodies, anti-apyrase antibodies, anti-NTPDase5 antibodies or anti-NPPase3 antibodies were used respectively, depending on the experiment, all the antibodies were purchased from Santa Cruz Biotechnology, Inc.). After washing with TBS, the membrane was incubated with the secondary antibody (anti-rabbit IgG Alkaline Phosphatase Conjugate) for 2 hours at room temperature. After removing the antibody, the alkaline phosphatase reagents (BCIP/NBT) were added.

### 2.7. NTPDases and NPPases expression analysis in ependymal cells

Total RNA was isolated from ependymocytes obtained by trypsynization of the ependymal surface with 0.25% trypsin and from fragments of brain tissue (positive control) frozen in liquid nitrogen. The reverse transcription was carried out with the use of First Strand cDNA Synthesis Kit. The primer sequences were designed as shown in Table 1.

**Table 1:** Primer sequences.

ENZYME	FORWARD	REVERSE
NTPDase1	5'-CTACCCCTTTGACTTCCAGG-3'	5'-GCACACTGGGAGTAAGGGC-3'

NTPDase2	5'-GGAGGCGAAGAGCAGG-3'	5'-TGGAGGCAGCCGCATGAAT-3'

NTPDase3	5'-AGCCTGGTCTCTTGGCTACA-3'	5'-ACCCCAGGCTGACTCTAAGC-3'

NTPDase5	5'-GTGAAAGGTGGCTCCCAA-3'	5'-CTTAGAGGTAGCCAAAGACTC-3'

NTPDase6	5'-ATGGGACCTTGCGGATGACGA-3'	5'-CCAAGCAACACATTCCATA-3'

NPPase3	5'-GTCAGAGCCATGAAATCCACT-3'	5'TCAGTACCATTTGAAGAAAGGATTTAGCTGTTCT-3'

The amplified cDNA fragments were electrophoresed in 1% agarose gel, stained with ethidium bromide and photographed under an ultraviolet light transilluminator.

### 2.8. Immunolocalization of nucleotide receptors on the surface of porcine brain ventricle lining

Fragments of intact ependymal surface obtained as indicated in section 2.2, were incubated with primary antibodies: anti-P2X_7_, anti-P2X_2 _and anti-P2Y_2 _(1 hour at 7°C) and subsequently with secondary antibodies conjugated with Alexa Fluor 488 (1 hour at 7°C). Preparations were analysed using confocal laser scanning microscope (Nikon Eclipse TE 300 CLSM) with Plan Apochromat 60 × objective. The Alexa Fluor 488 excitation with helium-neon laser (λ = 543 nm) resulted in red fluorescence of the fluorophore. The images were photographed with EZ 2000 Viewer for Confocal Microscope PCM 2000.

## 3. Results

### Nucleotides and nucleosides of porcine brain ventricular system

The cerebrospinal fluids (CSF) of seven healthy swines were used for qualitative and quantitative analyses of purines and pyrimidines. The selected animals were matched in respect to equal breed, age and farming conditions.

In porcine CSF we identified 8 purines and pirymidynes: ATP, ADP, AMP, Ado, GTP, GDP, UTP and UDP (Table 2). The values of standard deviation and median values similar to arithmetical mean are shown. We found that guanine nucleotides concentrations in analyzed CSF are significantly higher than adenine nucleotides concentrations. The [GTP]/[ATP] ratio is about 8:1 whereas the [GDP]/[ADP] ratio is 6:1. UTP was present only in 4 and UDP in 6 samples of analyzed CSF.

**Table 2:** Purines and pyrimidines concentration in porcine CSF.

	ATP	ADP	AMP	Ado	GTP	GDP	UTP	UDP
Purines concentration [μM]	1.04 ± 0.20*	3.71 ± 2.01	2.07 ± 0.88	2.20 ± 0.38	8.01 ± 3.79	20.35 ± 10.21	2.43 ± 1.3	0.32 ± 0.21

Median	0.98	4.25	1.92	2.23	7.67	21.00	0.99	0.18

*the results are expressed as mean ± standard deviation

### Immunochemical localization of nucleotide receptors on the surface of porcine brain ependyma

**Figure 1:**
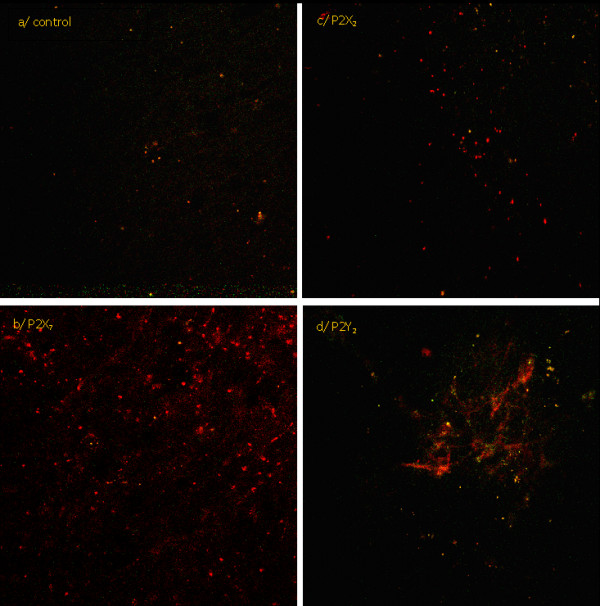
**Immunochemical localization of nucleotide receptors on the ependymal cells surface**. Signals from single anti-receptor antibodies (red), co-localized signals from antibodies (yellow), the auto-fluorescence of brain tissue (green).

We confirmed the presence of nucleotide receptors P2X_7_, P2X_2 _and P2Y_2 _in the ventricular system of porcine brain. These experiments were conducted on the unfixed samples of ependymal layer that significantly lowered the auto-fluorescence of brain tissue. P2X_7 _receptors are the most abundantly expressed nucleotide receptors on the ependymal cells (Figure 1b) whereas P2X_2 _receptors are less frequent and positive signals of anti-P2X_2 _antibodies are concentrated in specific regions of ependyma (Figure 1c). The widely expressed P2Y_2 _receptor in brain tissue is present on ependymal cells in streaks forming clusters (Figure 1d). The streaks of ependymal cells had been previously observed under electron microscope [39].

### The enzymes acting in nucleotides metabolism of porcine brain ventricular system

Nucleotides in CSF were hydrolysed by enzymes localized on the ependymal surface (ecto-nucleotidases) as well as by soluble enzymes present in CSF (exonucleotidases). These enzymes efficiently metabolised purine tri- and diphosphonucleotides (ATP > ADP > GTP > GDP). We also found the activity of 5'nucleotidase (Table 3) despite the fact that purine monophosphonucleotides and pyrimidynes were hydrolysed less efficiently.

**Table 3:** Substrate specificity of ependymal cells ecto-nucleotidases and exonucleotidases in CSF.

Substrate	Activity (%)	
	
	Ecto-nucleotidases*	Exo-nucleotidases**
**ATP**	100	100

**ADP**	95.1 ± 8.1	91.1 ± 6.2

**GTP**	60.0 ± 6.8	79.6 ± 7.6

**GDP**	42.3 ± 9.9	75.4 ± 7.9

**AMP**	12.2 ± 3.8	5.8 ± 3.2

**CTP**	5.1 ± 2.4	9.0 ± 2.9

**GMP**	4.8 ± 1.8	7.2 ± 3.2

**TTP**	3.7 ± 1.4	5.7 ± 3.5

**CDP**	3.3 ± 2.1	4.1 ± 2.1

**TDP**	0.8 ± 0.3	0.5 ± 0.6

*100% = 23 ± 6.3 nmol substrate/min^-1 ^× (cm^2^)^-1^

**100% = 20 ± 4.3 nmol substrate/min^-1 ^× mg protein^-1^

The reaction mixture consisted of 2 mM substrate, 2 mM Ca^2+^, 2 mM Mg^2+ ^in 50 mM Hepes-OH pH 7.4.

The kinetical analyses (inhibitors, ions and pH influence on the enzymes activity), Western Blotting and gene expression analyses helped to identify the nucleotidases present in brain ventricular system.

Ecto-enzymes associated with the ependymal cells surface and exo-enzymes present in CSF differed in their optimum pH or sensitivity to divalent ions. The different reaction products suggest that analysed enzymes belong to two distinct classes of hydrolases and kinases. The results are summarized in Table 4.

**Table 4:** The activity of nucleotidases of brain ventricular system under optimal pH conditions.

Incubation mixture		Ependymal ecto-enzymes activity (nmol × min^-1 ^× (cm^2^)^-1^)*			
**Ion**	**Inhibitor**	**ATP = > ADP**	**ATP = > AMP**	**ADP = > AMP**	**ADP = > ATP**
	
		**pH 7.5**	**pH 8.5**	**pH 7.5**	**pH 6.5**

Ca^2+^	Control (without inhibitor)	19.2 ± 1.1	20.1 ± 3.6	17.8 ± 3.5	11.3 ± 1.2
	
	Suramine	6.9 ± 1.2	17.2 ± 2.8	7.6 ± 1.3	11.2 ± 1.7
	
	Ap5A	16.5 ± 2.3	5.2 ± 0.9	17.7 ± 2.1	4.2 ± 1.0

Mg^2+^	Control (without inhibitor)	19.4 ± 1.9	20.4 ± 2.4	16.7 ± 2.0	18.5 ± 1.9
	
	Suramine	7.1 ± 2.0	19.4 ± 2.5	6.9 ± 1.5	18.2 ± 1.9
	
	Ap5A	21.0 ± 3.5	5.1 ± 1.1	16.5 ± 0.9	4.8 ± 0.9

**Incubation mixture**		CSF exo-enzymes activity (nmol × min^-1 ^× mg^-1^)*			

**Ion**	**Inhibitor**	**ATP = > ADP**	**ATP = > AMP**	**ADP = > AMP**	**ADP = > ATP**
	
		**pH 7.0**	**pH 8.0**	**pH 7.5**	**pH 6.5**

Ca^2+^	Control (without inhibitor)	21.1 ± 2.6	4.0 ± 0.8	18.1 ± 2.1	18.5 ± 2.1
	
	Suramine	10.9 ± 0.9	2.7 ± 0.9	11.2 ± 1.1	17.6 ± 1.2

	Ap5A	11.9 ± 1.2	2.8 ± 1.2	14.9 ± 0.8	4.6 ± 0.7
Mg^2+^	Control (without inhibitor)	23.0 ± 3.5	5.9 ± 2.3	25 ± 1.9	24.5 ± 2.1
	
	Suramine	13.2 ± 2.3	3.8 ± 1.6	15.2 ± 2.3	23.5 ± 3.2
	
	Ap5A	11.2 ± 1.6	4.4 ± 1.6	9.1 ± 0.1	5.3 ± 0.8

*expressed as concentration of reaction products

At pH 6.5 enzymes of CSF and ependymal ecto-enzymes use ADP to synthesize ATP. This reaction was activated by Mg^2+ ^ions, whereas Ap5A (kinase inhibitor) efficiently inhibited ATP synthesis, suggesting that nucleotide kinases are present in porcine CSF and on the surface of porcine brain ventricle lining.

#### Kinetical analyses of ependymal ecto-hydrolases

The values of optimal pH conditions for ATP to ADP hydrolysis at pH 7.5 were distinct from the optimal pH value 8.5 required for ATP to AMP hydrolysis. The ATP hydrolysis by ependymal ecto-enzymes at pH 7.5 was efficiently inhibited by suramine (NTPDases inhibitor) whereas Ap5A has no inhibitory effect. On the other hand, ATP to AMP hydrolysis at pH 8.5 was strongly inhibited by Ap5A (NPPases inhibitor).

ADP to AMP hydrolysis was most efficient at pH 7.5 and the hydrolytic activity decreased in the presence of suramine. These results indicate that ecto-NTPDase2, ecto-NPPase and enzyme ecto-NTPDase5, 6-like are the most active enzymes on the surface of ependymal cells.

#### Kinetical analyses of exohydrolases in cerebrospinal fluid

Further we found exo-enzymes in CSF that catalysed ATP to ADP hydrolysis at pH 7.0. ATP hydrolysis at this pH was efficiently inhibited by suramine and Ap5A. ATP to AMP was hydrolysed less efficiently - this process constituted only 20% of ATP to ADP hydrolysis.

The inhibitory effect of Ap5A on ADP to AMP hydrolytic activity was stronger in the presence of magnesium (64%) than calcium ions (40%). However, the inhibitory effect of suramine was stronger in the presence of calcium (49%) than magnesium ions (17%). These results indicate presence of soluble exoenzyme NTPDase2 activity in porcine CSF. The differences in the susceptibility of ADP to AMP hydrolysis to inhibitors, observed in the presence of Mg^2+ ^i Ca^2+^, indicate that the enzymes with NPPase-like activity (strong inhibition with Ap5A in presence of Mg^2+ ^[36,37]) and NTPDase5 or NTPDase6 activity (strong inhibition with suramine in presence of Ca^2+ ^[35]) are present in CSF of swine.

### Molecular identification of brain ventricular system nucleotidases

**Figure 2:**
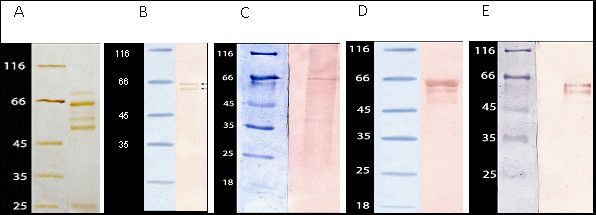
**Molecular analysis of proteins present in porcine CSF**. A/Polyacrylamide gel electrophoresis stained with silver, B/Western Blotting with anti-NTPDase1 (CD39) antibodies, C/Western Blotting with anti-apyrase from *Arabidopsis thaliana *antibodies, D/Western Blotting with anti-NTPDase5 antibodies, E/Western Blotting with anti-NPPase3 antibodies.

The enzymatic proteins present in porcine CSF were detected and identified. The results of SDS-PAGE electrophoresis and Western Blot analyses are shown in Figure 2.

The presence of enzymes from NTPDase and NPPase families in porcine CSF was confirmed by their molecular mass (Figure 2A) as well as Western Blotting analyses. However, the precise identification of the enzymes was impossible. The molecular weight of proteins interacting with applied anti-NTPDases primary antibodies was about 64 kDa, 62 kDa and 58 kDa respectively (Figure 2B, D). It is consistent with the molecular weight of soluble NTPDases of blood vessels [25,40] and apyrase of *A. thaliana *(Figure 2C). The proteins interacting with anti-NPPases antibodies reflected molecular weight of about 52 kDa and 58 kDa (Figure 2D, E). These data are in good agreement with molecular weight of soluble NPPases found in literature [41-48].

The above experiments on gene expression indicate, that genes coding the soluble NPPase3 (Figure 3.1E), NTPDase5 (Figure 3.3E) and NTPDase6 (Figure 3.5E) as well as NTPDase2 (Figure 3.2E) are expressed in ependymal cells.

**Figure 3:**
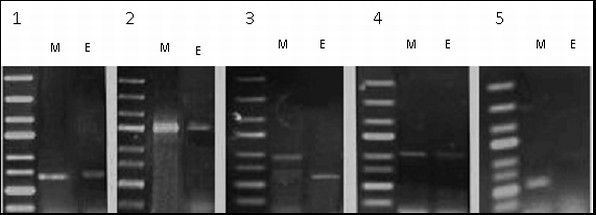
**Expression analyses of ependymal cells nucleotidases**. PCR reaction products: **M **- with the mRNA isolated from the whole brain tissue used as template, **E **- with the mRNA isolated from ependymal layer cells used as template. The primers were prepared for: 1/NPPase3; 2/NTPDase2; 3/NTPDase5; 4/NTPDase6; 5/NTPDase1.

PCR products obtained with the use of primers for NTPDase5 from porcine brain cells and ependymal cells were different in respect of their size (Figure 3.3), suggesting distinct splicing of NTPDase5 in ependymal cells than its splicing in the brain.

Our results show that there is no NTPDase1 expression in ependymal cells of porcine brain (Figure 3.5E), whereas it is expressed in brain (positive control, Figure 3.5M). The absence of NTPDase1 expression in ependymal cells may constitute the negative control used for the identification of these cells.

The presence of NTPDase3 in ventricular system of porcine brain cannot be confirmed by PCR reactions. However, in the literature there are data concerning the NTPDase3 expression in mammalian and human brain [49,50].

## 4. Discussion

The results presented in this paper for the first time demonstrate the presence of all three elements of purinergic signaling in the swine (*Sus scrofa domestica*) brain ventricular system. Several previous studies showed the correlation between concentration of nucleotides, nucleosides and purine/pyrimidine bases in extracellular spaces of brain tissue and cerebrospinal fluid [1-4,12-14]. However, previous experiments on ventricular system metabolism were conducted in rodents [5,15,39,51-56]. Rodents brain tissue retained quite well functioning repair processes because of high differentiating potential of neuronal stem cells present in the subventricular zone [57,58]. In the contrary to other mammalian brains, the brain of rodents responds differently to the non-physiological conditions, like ischemia or hypoxia, that damage cells of the central nervous system [54,55,59].

### Nucleotides and nucleosides of porcine brain ventricular system

The presence of ATP, ADP and other tri- and diphosphonucleotides in human CSF was indicated for the first time by Czarnecka et al. [32]. The rabbit CSF also possesses a wide range of various compounds including adenosine, guanosine, uridine, cytidine, inosine, tymidine, deoxycytidine, deoxyuridine, hipoxanthine, xanthine and uric acid. The concentration of these compounds was tested in normal physiological conditions, during starving and liver damage [60,61]. Similarly, rodents were also shown to carry nucleotides in their CSF [60,61].

Our experiments indicated that similar to the concentration of purines in human tissue fluids [1-4,12-14] median values of ATP, ADP, AMP, GTP, GDP, UTP, UDP and adenosine in swine CSF ranged from 1 to 2 μM.

We further found that concentrations of guanine nucleotides in analyzed CSF are significantly (about 8 times) higher than adenine nucleotides concentrations. The high concentrations of guanine nucleotides in porcine CSF are probably due to the absence of ecto- and exo-enzymes capable of specific guanine nucleotides hydrolysis. The maintaining of high level of guanine nucleotides suggests an important role for these nucleotides in ventricular system physiology. Though the precise function of guanine nucleotides is still unclear, however. More recent studies suggest the participation of GTP and guanosine in metabotropic (P2Y) receptors activation during regulation of ATP and adenosine exocytosis [17,18,62-66]. Additionally, the latest data indicate that guanine nuclotides can act as trophic agents [18].

The correlation between concentration of nucleotides, nucleosides and purine/pyrimidine bases in extracellular spaces of brain tissue on one hand and in cerebrospinal fluid on the other, suggests that changes in CSF composition may reflect changes in the brain condition [1-4,12-14]. Numerous authors claim that brain hypoxia results in ATP degradation and in signifficant increase of degradation products in CNS cells. Some of the products, like adenosine, can be released in cerebrospinal fluid [17,18,67-69]. On the other hand, the changes in the CNS cells metabolism can induce intensified exocytosis of ATP and ADP [19,68]. Damaged brain cells constitute another source of purines and pyrimidines [19].

### Nucleotide receptors

The nucleotide receptors were found in all organs and tissues examined so far [19]. In rodents ventricular system their existence was confirmed immunochemically [15,16]. The PCR method allowed to detect mRNA of nucleotide receptors in cells of choroid plexus and spinal cord neurons that come in contact with CSF in rodents [15,70]. However, these studies did not successfully determine the exact amount and localization of these receptors on the cell surface. The results presented in this paper indicate that nucleotide receptors P2X_2_, P2X_7 _and P2Y_2 _are expressed on the ependymocytes of lateral ventricle of porcine brain. Their amount and distribution has been found to be diverse. The P2X_7 _receptors connected with inflammatory reactions and pain [71] were predominantly expressed on the analysed tissue fragments. The density of P2X_2 _i P2Y_2 _receptors was low, whereas P2Y receptors were distributed irregularly in form of streaks and P2X receptors were scattered on the ependymocytes surface. The presence of nucleotide receptors in the areas contacting with CSF suggests the possibility of communication between ependymal cells and cerebrospinal fluid via nucleotides. Furthermore, high density of P2X_7 _receptors indicates that ventricular system plays a key role in inflammatory processes in central nervous system since in recent years, the role of ATP and P2X_7 _receptors in these processes has been recognised [72-74]. It was also found that injury inflicted on rat brain by heme injection resulted in increased interleukine concentration in CSF [75]. We detected the elevated concentration of ATP in CSF of patients with brain stroke (unpublished data). The results discussed above confirm the thesis about cross-talking between brain and ventricular system functioning in physiological as well as pathological conditions.

Nucleotide receptors, like all the receptors of neurotransmitters in the ventricular system, may also be involved in the regulation of cilliae movement [5-11], important for CSF circulation.

### The enzymes acting in nucleotides metabolism of porcine brain ventricular system

The enzymes associated with ependyma and CSF hydrolysed adenosine tri- and diphosphonucleotides effectively while guanine nucleotides were not hydrolysed as efficiently and pyrimidynes were not metabolised at all. Similar substrate specificity was found for soluble nucleotidases in rat CSF [25]. The nucleotidases in ventricular system, like the majority of so far characterised NTPDases [EC 3.6.1.5] and NPPases [EC 3.6.1.9], acted optimally in alkaline conditions at pH 7.5-8.0 [25,29,42-49]. These enzymes were activated by divalent ions, preferably by Ca^2+ ^and Mg^2+^. Moreover, the susceptibility to inhibitors of ecto-nucleotidases and kinases (suramine and Ap5A) and alkaline phosphatases (levamisole) indicates that ecto-NTPDases as well as NPPases are active on the ependymocytes. We also realize the presence of nucleotide kinases in porcine CSF and on the surface of porcine brain ventricle lining. Inconsiderable influence of levamisole on nucleotides hydrolysis (data not shown) excludes the presence of membranous alkaline phosphatases. In summary, ecto- and exonucleotidases from NTPDase and NPPase family as well as adenylate kinase participate in nucleotides metabolism in ventricular system of porcine brain.

**Figure 4:**
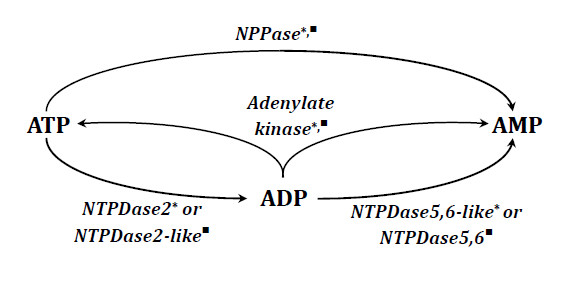
The sequence of conversions and the enzymes engaged in purines metabolism on the surface of ependymal cells (marked as *) and in the cerebrospinal fluid (marked as ^■^) of porcine brain; detailed description in the text.

The adenylate kinase (AK), ectoNTPDase2, NPPase and 5'-nucleotidase activities were demonstrated on the surface of ependymal cells (Figure 4). Kinetical analyses failed to verify activity of NTPDase1 and/or 3. The PCR method also confirmed the absence of NTPDase1 in ventricular system of swine. Unexpectedly, in Western Blotting anti-NTPDase1 antibodies reacted with protein of molecular mass characteristic for soluble NTPDases, which allowed us to conclude that they reacted non-specifically with NTPDase5 protein.

In porcine CSF we also detected the activity of NPPase and nucleotidases similar to NTPDase2 and NTPDase 5 or 6 (Figure 4). NPPase of CSF is inhibited by Ap5A. The enzyme with similar properties was found in rat CSF [76]. Western blot analysis indicated that the proteins interacting with anti-NPPases antibodies have molecular weight of about 52 kDa and 58 kDa. These data are in accordance with molecular weight of soluble NPPases given in literature [41-48]. One of these proteins is NPPase3, analogous to ectoNTPDases in blood vessels [76], the difference in the molecular weight (about 6 kDa) suggests that the second protein can be NPPase3 that might have been detached from the membrane and released to CSF.

We also detected the soluble form of NTPDase hydrolysing ATP and less efficiently ADP, which is inhibited by suramine and not by Ap5A. However, the soluble NTPDases described so far had rather high specificity to diphosphonucleosides and low specificity to triphosphonucleosides [40,77,78]. We also found the adenylate kinase activity in cerebrospinal fluid of swine. We detected low 5'-nucleotidase and adenosine deaminase activities in porcine CSF.

Mainly neuronal NTPDases are involved in release of the agonists from nucleotide receptors [25,29,50]. The end product of extracellular ATP and ADP metabolism in ventricular system is always AMP. Blood vessel NTPDase1, 2 and 3 activity and neuronal ecto-NTPDases also produced AMP [25,29,50]. In opposition to that, we did not find NTPDase 1 and 2 in ventricular system. The product of the above reactions - extracellular AMP is further hydrolysed by ependymal 5'-nucleotidase to adenosine that is known for its neuroprotective functions [67,69]. The activity of 5'-nucleotidase was found both on ependymal surface and CSF, however in CSF the hydrolysis of AMP is due to unspecific phosphatase activity.

The results of previous studies suggested that NTPDases participate in CSF nucleotides metabolism [25,50]. Nucleotidase activity using di- and triphosphonucleosides was detected in rat CSF, however the precise identification of these NTPDases was imposible [76]. There is little information about participation of other enzymes, for example exo-nucleotidases, in nucleotide metabolism. Previous experiments indicated the presence of NPPase2 and 3 on chondroid plexus cells [79].

Extracellular nucleotides including ATP, ADP, UTP and UDP are signaling molecules involved in the regulation of metabolic processes throughout the nervous system [26,27]. In recent years the particular attention has been paid to the role of ATP and adenosine in inflammatory reactions. ATP is considered to be specific "danger signal". Released to the extracellular space ATP informs about pathological conditions and damage of cells and activates immunological response [80]. The presented results not only confirmed the presence of all elements of purinergic signaling in the mammalian brain ventricular system, but also demonstrated numerous P2X_7 _receptors expressed on the surface of ependymal cells. These observations suggest that the ventricular system is responsible for initiating the inflammation reactions triggered off by the damage of brain cells. Relevance of these observations is elevated due to the fact that increased concentration of ATP, ADP and GTP in CSF of patients with brain stroke may be connected with higher death risk (unpublished data).

## 5. Declaration of competing interests

The authors declare that they have no competing interests.

## 6. Authors' contributions

JC carried out the biochemical analyses and molecular studies and drafted the manuscript, KR participated in the biochemical analyses and drafted the manuscript, AJ have been involved in the collection and preparation of animal material, DS carried out the immunochemical experiments, MK contributed conception and design of the study, analysis and interpretation of the data and drafting the manuscript. All authors read and approved the final manuscript.
